# Supplementary material for: Evaluation of the role of whiB6 and kdpDE in the dominant multidrug-resistant clone Mycobacterium tuberculosis B0/W148
Source: Microbiol Spectr. 2025 May 22;13(7):e03224-24. doi: 10.1128/spectrum.03224-24 (PMC12210973; doi:10.1128/spectrum.03224-24)
Supplement: Table S4 and Fig. S1 to S — Primer sequences; Functional annotation and categorization of genes detected as differentially expressed in the whiB6 and kdpDE genotypes relative to H37Rv; Original Western blots corresponding to Fig. 3D; Virulence evaluation of whiB6 T51P and kdpDE ΔCA mutations in BALB/c mice. [file spectrum.03224-24-s0001.docx]

**Fig. S1. Functional annotation and categorization of genes detected as differentially expressed in the *whiB6* genotypes relative to H37Rv.** Gene overlap is depicted as a proportion of differentially expressed genes belonging to each functional category relative to the total number of genes detected as differentially expressed in each *whiB6* genotype as compared to H37Rv (x-axis, n). Total number of genes annotated within each functional category is indicated (y-axis, N).


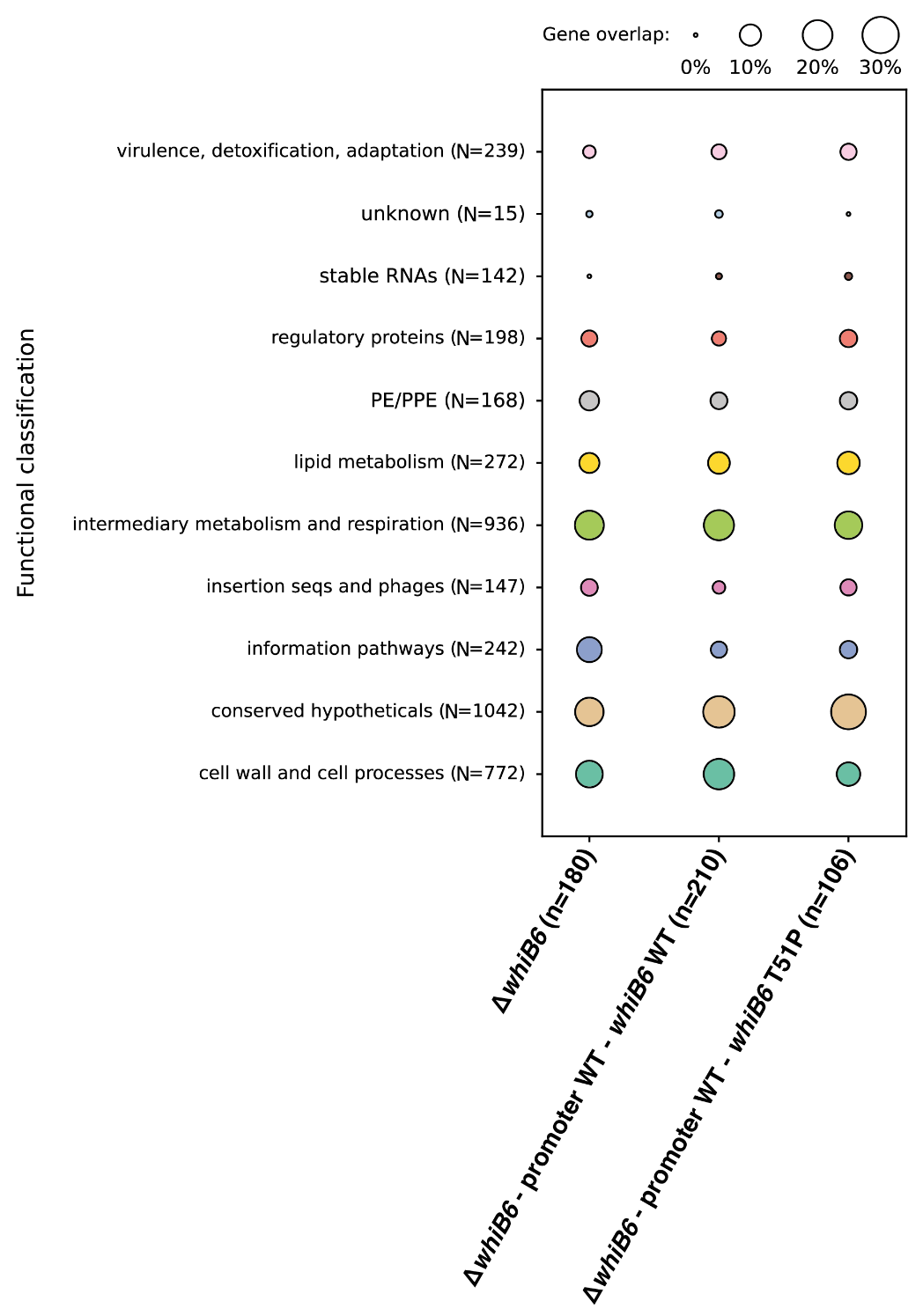


**Fig. S2. Original Western blots corresponding to Figure 3D.** Forensic analysis of Figure 3D has identified small straight lines between samples H37Rv and H37Rv∆RD1, and between ∆*whiB6* – promoter G insertion – *whib6* WT and ∆*whiB6* – promoter WT – *whib6* WT, which might suggest splicing or assembly of the different blots in the figure. This is however not the case, as no stitching or assembly are reported in the original images and notes from the lab book as shown below. The observed lines likely result from the migration of an impurity in the gel.


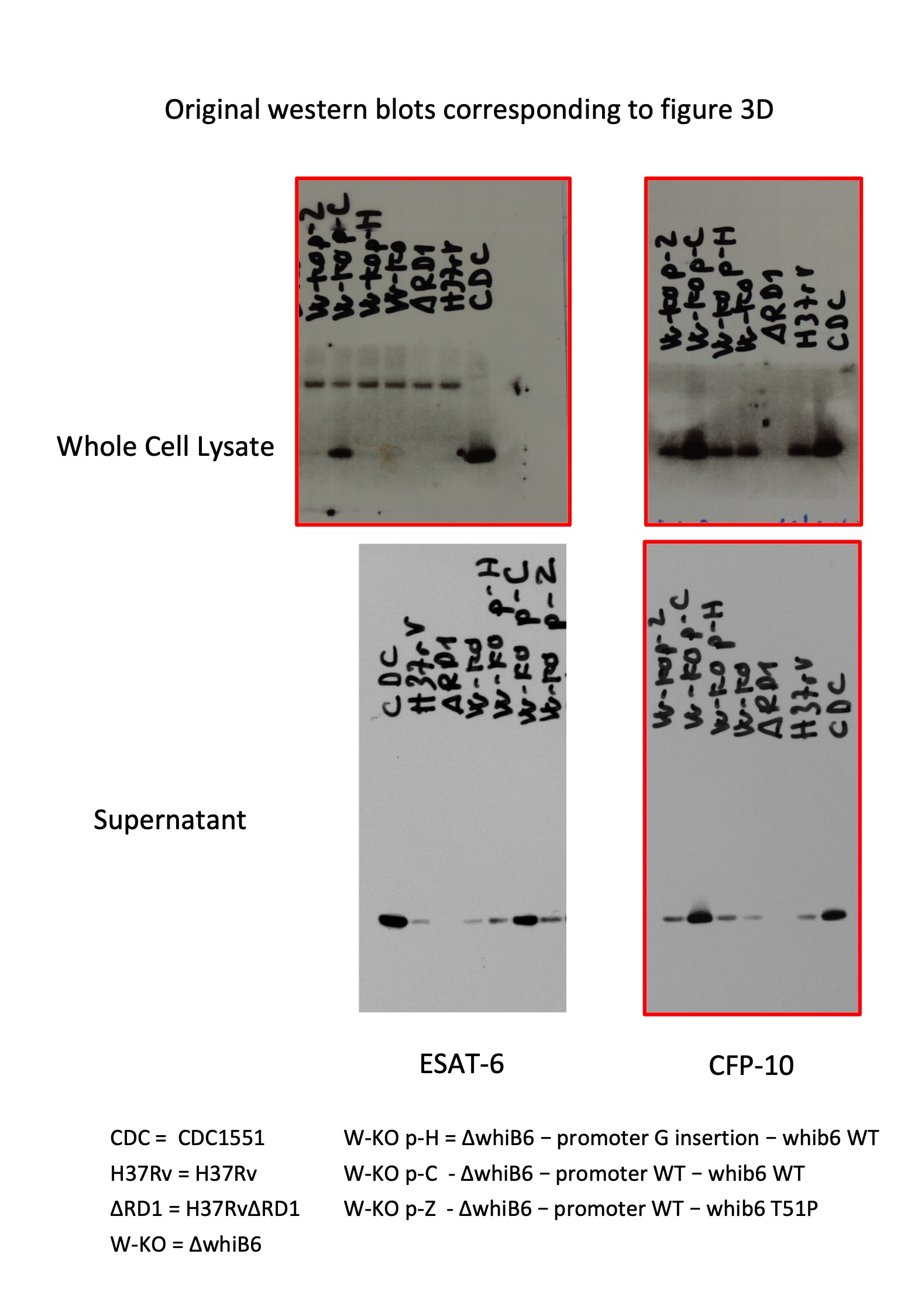


Where:

| CDC = CDC1551 | W-KO = ∆*whiB6* |
| --- | --- |
| H37rv = H37Rv | W-KO p-H = ∆*whiB6* – promoter G insertion – *whiB6* WT |
| ∆DR1 = H37Rv∆RD1 | W-KO p-C = ∆*whiB6* – promoter WT – *whiB6* WT |
|  | W-KO p-Z = ∆*whiB6* – promoter WT – *whiB6* T51P |

Note that the Western blots in red frame are in the inverse orientation as compared to the Figure 3D and the ESAT-6 Western blot of the supernatant.

**Fig. S3. Functional annotation and categorization of genes detected as differentially expressed in the *kdpDE* genotypes relative to H37Rv in the presence of potassium.** Gene overlap is depicted as a proportion of differentially expressed genes belonging to each functional category relative to the total number of genes detected as differentially expressed in each *kdpDE* genotype as compared to H37Rv during normal growth condition (x-axis, n). Total number of genes annotated within each functional category is indicated (y-axis, N).


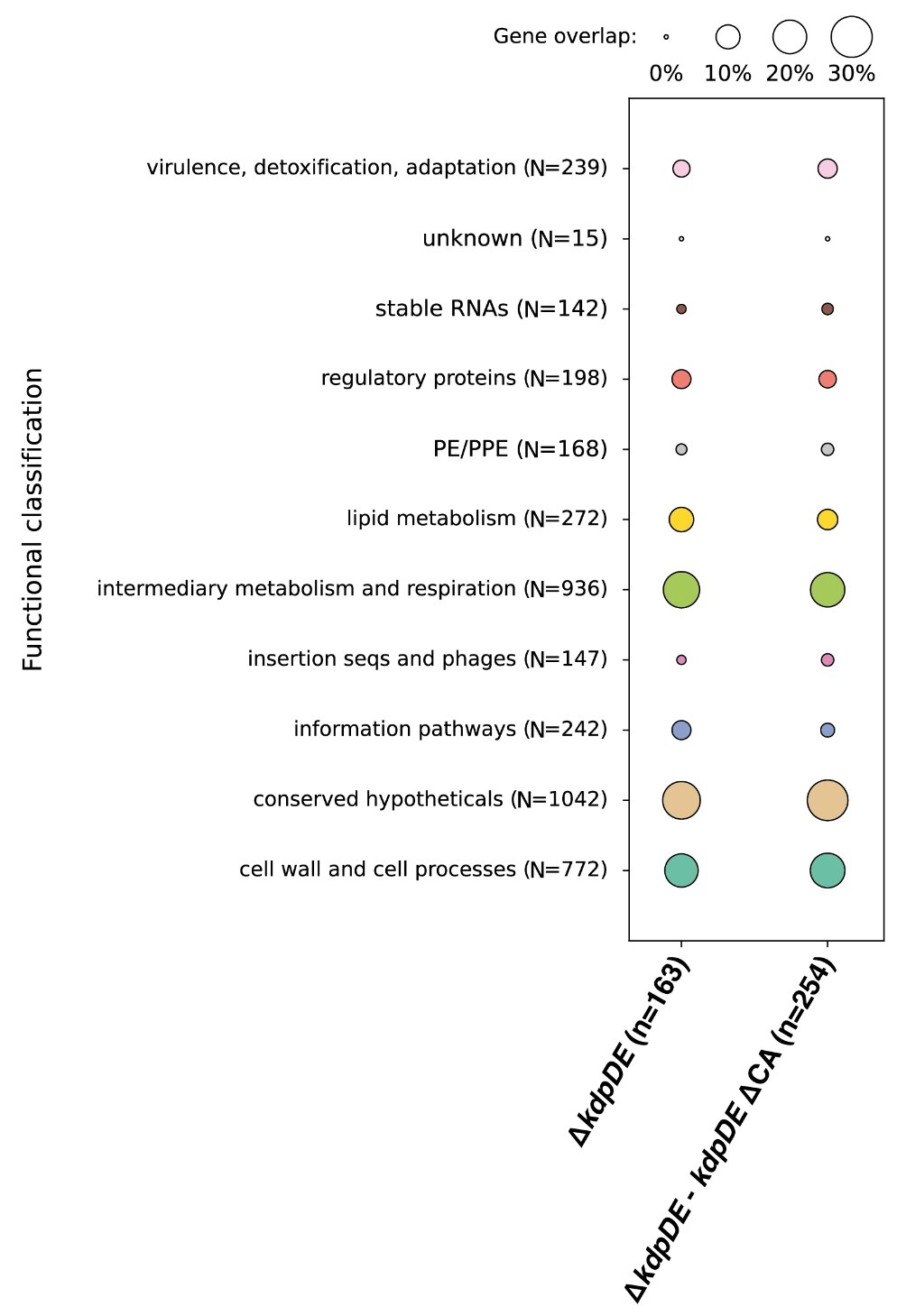


**Fig. S4. Functional annotation and categorization of genes detected as differentially expressed in the *kdpDE* genotypes relative to H37Rv in the absence of potassium.** Gene overlap is depicted as a proportion of differentially expressed genes belonging to each functional category relative to the total number of genes detected as differentially expressed in each *kdpDE* genotype as compared to H37Rv upon potassium depletion (x-axis, n). Total number of genes annotated within each functional category is indicated (y-axis, N).


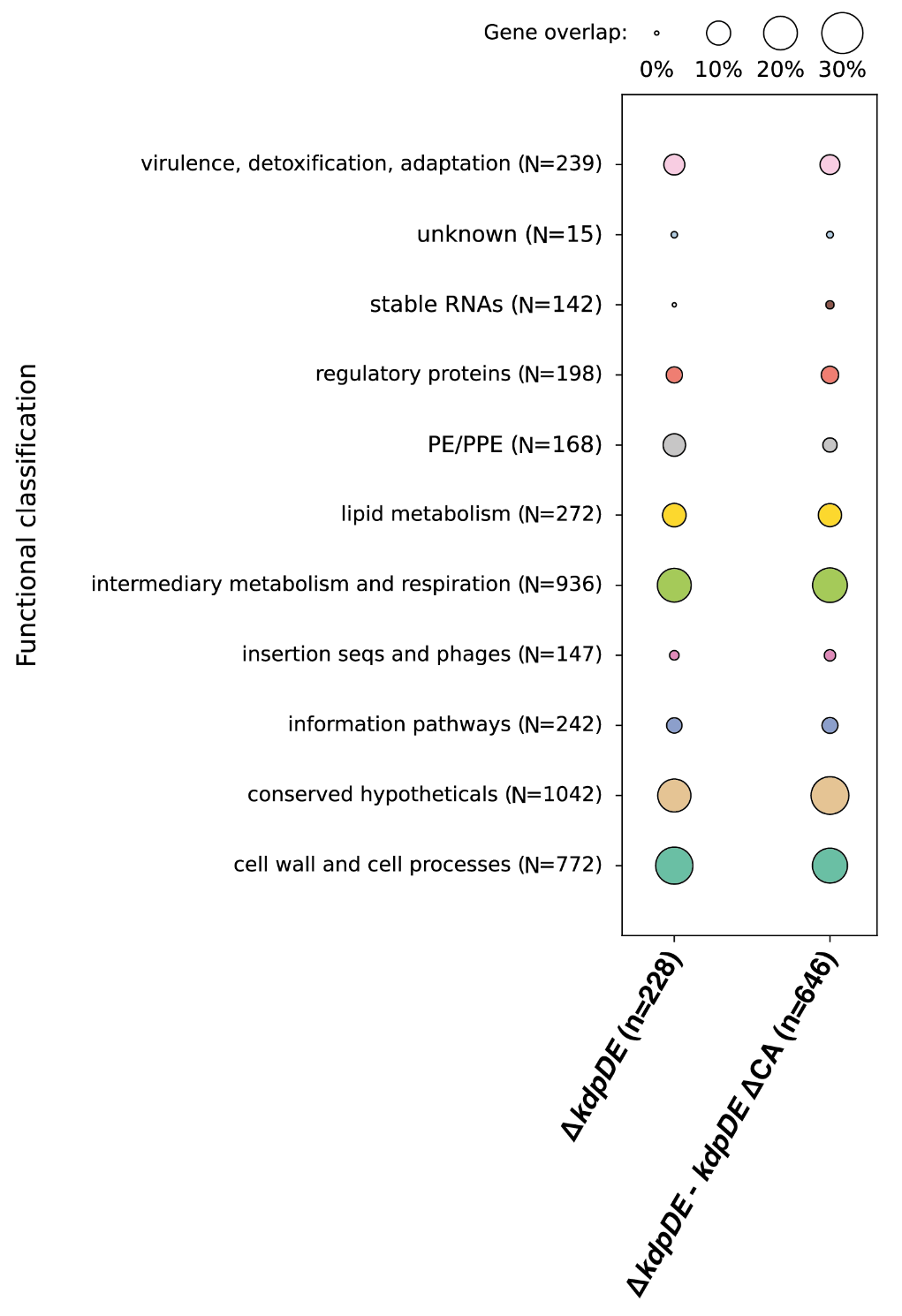


**Fig. S5. Virulence evaluation of *whiB6* T51P and *kdpDE* ΔCA mutations in BALB/c mice.** (A) Time course after mouse infection with H37Rv, the ∆*whiB6* mutant strain, and the promoter G insertion - *whiB6* WT, the promoter WT - *whiB6* WT, and the promoter WT - *whiB6* T51P complemented strains. (B) Time course after mouse infection with H37Rv, the ∆*kdpDE* mutant strain, and both *kdpDE* WT and ∆CA complemented strains. Recovery of the bacteria is enumerated by CFU per lung at 1, 30, 60 days after injection. Means for 5 mice at day 1 and 30 or for 10 mice at day 60 are shown with one point representing a mouse. Extra points correspond to mice killed between day 30 and day 60 due to probable otitis; they are attributed to day 30 or day 60 depending on the closest date.


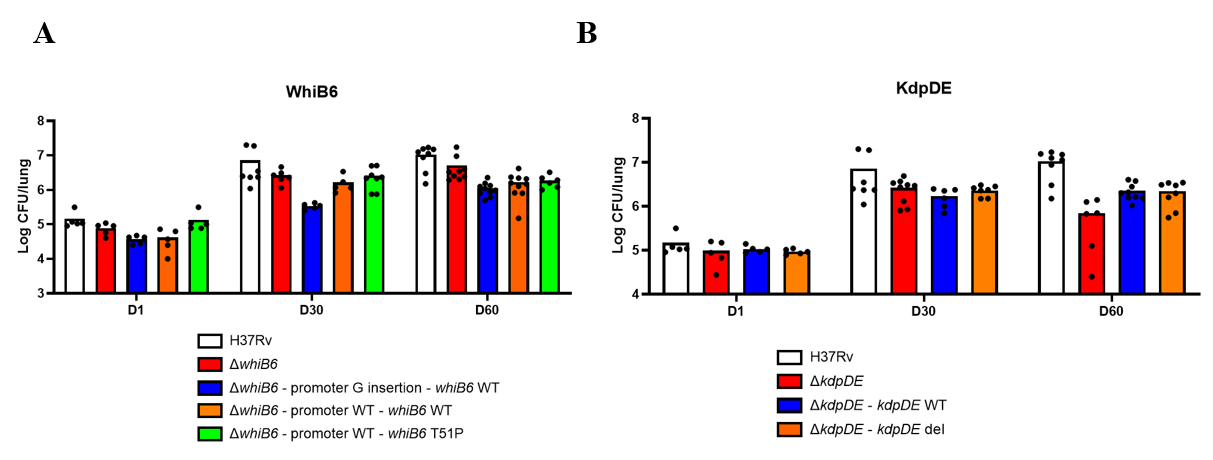


**Table S4. Primer sequences.**

|  | **Name** | **Sequence** |
| --- | --- | --- |
|  | Construction of Mtb H37Rv∆*whiB6* | |
| **1** | Up-region_*whiB6*_Forward | 5’-GATTCGGACGGACACGCCGA-3’ |
| **2** | Up-region_*whiB6*_Reverse | 5’-ttcgttttatttgatgcctgTCAGGCCGGGCGCGGGCATT-3’ |
| **3** | Down-region_*whiB6*_Forward | 5’-agttttcgttccactgagcgTAACCATAGCGATGCAACAG-3’ |
| **4** | Down-region_*whiB6*_Reverse | 5’-CCAATGATCGCGACACCGCG-3’ |
|  | Construction of pYUB412_promoter insertion G - *whiB6* WT, pYUB412_promoter WT - *whiB6* WT, pYUB412_promoter WT - *whiB6* T51P | |
| **5** | WhiB6_EcoRV_Forward | 5’-GTAGGATATCGACGCCTAACCGTTGCACCCTTCT-3’ |
| **6** | WhiB6_AseI_Reverse | 5’-CGGCATTAATCTTCTCATGCCGATTGGGCAGACA-3’ |
|  | Construction of Mtb H37Rv∆*kdpDE* | |
| **7** | Up-region_*kdpDE*_del_Forward | 5’-cgtctaagaaaccTGAAGGCGACATGGTCGG-3’ |
| **8** | Up-region_*kdpDE*_del_Reverse | 5’-tcgactgagccttGTGCACAAGAATCGAGAGG-3’ |
| **9** | Down-region_*kdpDE*_del_Forward | 5’-cctgcatgaccaaTTTCCGCTGGGAGCGGATG-3’ |
| **10** | Down-region_*kdpDE*_del_Reverse | 5’-gacttcagagcttTCGTGGTCGAAGAGTTGGC-3’ |
|  | Construction of pYUB412_*kdpDE* WT plasmid | |
| **11** | *KdpDE*_XbaI_Forward | 5’-ccctctagaGTTGTCGACCGTAGTCAT-3’ |
| **12** | *KdpDE*_XbaI_Reverse | 5’-ccctctagaGTCACATCTGCCACACG-3’ |
|  | Construction of pYUB412_*kdpDE* del plasmid | |
| **13** | *KdpDE*_pYUB412_Forward | 5’-GGGCGGCGGGCTCAGTGGTGATCG-3’ |
| **14** | *KdpDE*_pYUB412_Reverse | 5’-CGATCACCACTGAGCCCGCCGCCC-3’ |
